# An online peak extraction algorithm for ion mobility spectrometry data

**DOI:** 10.1186/s13015-015-0045-5

**Published:** 2015-05-13

**Authors:** Dominik Kopczynski, Sven Rahmann

**Affiliations:** Bioinformatics for High-Throughput Technologies, Computer Science XI, and Collaborative Research Center SFB 876, TU Dortmund, Dortmund, Germany; Genome Informatics, Institute of Human Genetics, University Hospital Essen, University of Duisburg-Essen, Essen, Germany

**Keywords:** Ion mobility spectrometry, Peak detection, Automated data analysis, Online analysis

## Abstract

Ion mobility (IM) spectrometry (IMS), coupled with multi-capillary columns (MCCs), has been gaining importance for biotechnological and medical applications because of its ability to detect and quantify volatile organic compounds (VOC) at low concentrations in the air or in exhaled breath at ambient pressure and temperature. Ongoing miniaturization of spectrometers creates the need for reliable data analysis on-the-fly in small embedded low-power devices. We present the first fully automated online peak extraction method for MCC/IMS measurements consisting of several thousand individual spectra. Each individual spectrum is processed as it arrives, removing the need to store the measurement before starting the analysis, as is currently the state of the art. Thus the analysis device can be an inexpensive low-power system such as the Raspberry Pi.

The key idea is to extract one-dimensional peak models (with four parameters) from each spectrum and then merge these into peak chains and finally two-dimensional peak models. We describe the different algorithmic steps in detail and evaluate the online method against state-of-the-art peak extraction methods.

## Introduction

Ion mobility (IM) spectrometry (IMS), coupled with multi-capillary columns (MCCs), MCC/IMS for short, has been gaining importance for biotechnological and medical applications. With MCC/IMS, one can measure the presence and concentration of volatile organic compounds (VOCs) in the air or in exhaled breath with high sensitivity. In contrast to other technologies, such as mass spectrometry coupled with gas chromatography (GC/MS), MCC/IMS works at ambient pressure and temperature. Several diseases like chronic obstructive pulmonary disease (COPD) [1], sarcoidosis [2] or lung cancer [3] can potentially be diagnosed early with MCC/IMS technology. IMS is also used for the detection of drugs [4] and explosives [5]. Constant monitoring of VOC levels is of interest in biotechnology, e.g., for watching fermenters with yeast producing desired compounds [6] and in medicine, e.g., monitoring propofol levels in the exhaled breath of patients during surgery [7].

IMS technology is moving towards miniaturization and small mobile devices. This creates new challenges for data analysis: The analysis should be possible *within* the measuring device without requiring additional hardware like an external laptop or a compute server. Ideally, the spectra can be processed on a small embedded chip or small device like a Raspberry Pi or similar hardware with restricted resources. Algorithms in small mobile hardware face constraints, such as the need to use little energy (hence little random access memory), while maintaining prescribed time constraints.

The basis of MCC/IMS analysis is *peak extraction*, by which we mean a representation of all high-intensity regions (peaks) in the measurement by using a few descriptive parameters per peak instead of the full measurement data. State-of-the-art software (like IPHEx [8], Visual Now [9], PEAX [10]) only extracts peaks when the whole measurement is available, which may take up to 10 minutes because of the pre-separation of the analytes in the MCC. Our own PEAX software in fact defines modular pipelines for fully automatic peak extraction and compares favorably with a human domain expert doing the same work manually when presented with a whole MCC/IMS measurement. However, storing the whole measurement is not desirable or possible when the memory and CPU power is restricted. Here we introduce a method to extract peaks and estimate a parametric representation while the measurement is being captured. This is called *online peak extraction*, and this article presents the first algorithm for this purpose on MCC/IMS data. An extended abstract of this work has been published at WABI’14 [11].

Section ‘Background’ introduces the necessary background on the data produced by an MCC/IMS experiment, on peak modeling and on optimization methods. The basic idea of our algorithm is to process each IM spectrum as soon as it arrives (and before the next one arrives). After appropriate pre-processing including denoising and baseline correction described in Section ‘Denoising and baseline correction’, the single spectra are reduced into a mixture of parametric one-dimensional peak models, described in Section ‘Reducing a spectrum to peak models’. Accordingly, in Section ‘Aligning consecutive spectrum peak lists’ the approach of connecting models from two subsequent spectra into peak chains is explained. The main challenge is then to merge the peak chains into two-dimensional peak models, described in Section‘Estimating 2-D peak models’. In Section ‘Peak clustering’ we introduce a novel approach for clustering peaks among several measurements e.g. for time series. An evaluation of our approach is presented in Section ‘Evaluation’ including a listing of all settings of the MCC/IMS as well as an explanation of all adjustable parameters, while Section ‘Discussion and conclusion’ contains a concluding discussion.

## Background

Ion mobility spectrometers and their functions are well documented [12], and we do not go into technical details. Instead, we characterize the data generated by an MCC/IMS experiment (Section ‘Data from MCC/IMS measurements’). In Section ‘Peak models’ we describe a previously used parametric peak model, and in Section ‘Optimization methods’ we review two optimization methods that are being used as subroutines in this work.

### Data from MCC/IMS measurements

In an MCC/IMS experiment, a mixture of several unknown volatile organic compounds (VOCs) is separated in two dimensions: first by retention time *r* in the MCC (the time required for a particular compound to pass through the MCC) and second by drift time *d* through the IM spectrometer. Instead of the drift time itself, a quantity normalized for pressure and temperature called the *inverse reduced mobility* (IRM) *t* is used to compare spectra taken under different or changing conditions. Thus we obtain a time series of IM spectra (one spectrum each 100 ms at each retention time point), and each spectrum is a vector of ion concentrations (measured by voltage change on a Faraday plate) at each IRM.

**Figure 1:**
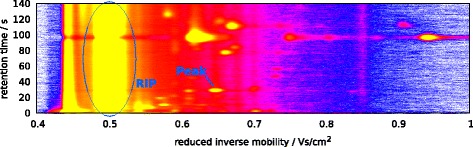
Visualization of a raw measurement (IMSC) as a heat map; signal color: white (lowest) < blue < purple < red < yellow (highest). The constantly present reactant ion peak (RIP) with mode at 0.48 Vs/cm^2^ and exemplarily one VOC peak are annotated.

Let *R* be the set of (equidistant) retention time points and let *T* be the set of (equidistant) IRMs where a measurement is made. If *D* is the corresponding set of drift times (each 1/250000 second for 50 ms, that is 12 500 time points), there exists a constant *C*_t|d_>0 depending on external conditions [12] such that *T*=*C*_t|d_·*D*. Then the data is an |*R*|×|*T*| matrix *S*=(*S*_*r*,*t*_) of measured ion intensities, which we call an *IM spectrum-chromatogram* (IMSC). The matrix can be visualized as a heat map (Figure 1). A row of *S* is a *spectrum*, while a column of *S* is a *chromatogram*.

Areas of high intensity in *S* are called peaks, and our goal is to discover them and to describe them by parametric models. Comparing peak coordinates with reference databases may reveal the identity of the corresponding compound. A peak caused by a VOC occurs over several IM spectra. We mention some properties of MCC/IMS data that complicate the analysis.

**Figure 2:**
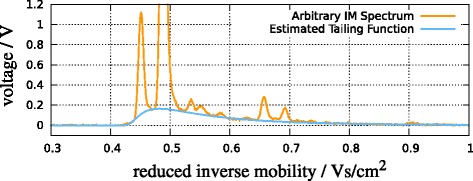
A spectrum and its estimated tailing function.

- An IM spectrometer uses an ionized carrier gas. These ions are present in every spectrum in addition to the analyte ions, and they create the *reactant ion peak* (RIP). In the whole IMSC it is present as high-intensity chromatogram at a specific IRM (Figure 1). When no analytes are injected into the device, the spectra contain only the RIP and are called *RIP-only spectra*.
- Every spectrum contains a tailing of the RIP, so the RIP is right-skewed (Figure 2). To extract peaks, the effect of the RIP and its tailing must be estimated and removed.
- At higher concentrations, compounds can form dimer ions, and one may observe both the monomer and dimer peak from one compound. This means that there is not necessarily a one-to-one correspondence between peaks and compounds, and our work focuses on peak detection, not compound identification.
- An IM spectrometer may operate in positive or negative mode, depending on which type of ions (positive or negative) one wants to detect. In either case, signals are reported in positive units. All experiments described here were done in positive mode.

### Peak models

For our purpose of analyzing MCC/IMS measurements, a peak is characterized by the following assumptions.

#### **Assumptions1**.

An *n*-dimensional peak *P* is a product of *n* log-concave functions with two inflection points in each dimension. The peak width at half height *ω*_1/2,*i*_ can be calculated with respect to the mode for each dimension *i*. At its mode (*m*_1_,…,*m*_*n*_), peak *P* exceeds the average background noise level by a certain factor times the standard deviation of the noise.

For MCC/IMS measurements, we have *n*=2 dimensions, and in both retention time dimension and IRM dimension, we use the shifted Inverse Gaussian distribution *g* [13] as peak model function:

(1) $$\begin{array}{*{20}l} & g(x; \mu, \lambda, o) := \frac{1[x > o]}{\sqrt{2\pi}} \cdot  \\ & \cdot \sqrt{\frac{\lambda}{(x - o)^{3}}} \,\cdot\, \exp \left(-\frac{\lambda \left((x - o)-\mu \right)^{2}}{2 \mu^{2} (x - o)} \right).  \end{array} $$

Its parameters are the shift (or offset) *o*, the relative mean *μ*>0 (to the right of *o*) and the shape parameter *λ*>0. A peak is then given as the product of two shifted Inverse Gaussians, scaled by a volume factor *v*, i.e., by seven parameters; so the density function of a peak is *p*(*r*,*t*):=*v*·*g*(*r*,*μ*_r_,*λ*_r_,*o*_r_)·*g*(*t*,*μ*_t_,*λ*_t_,*o*_t_) for all *r*∈*R*,*t*∈*T*.

Since the parameters *μ*,*λ*,*o* of a shifted Inverse Gaussian may be different even though the resulting distributions have a similar shape, it is more intuitive to describe the shifted Inverse Gaussian in terms of three different *descriptors*: the (absolute) mean *μ*^′^=*o*+*μ*, the standard deviation *σ* and the mode *m*. There is a bijection between (*μ*,*λ*,*o*) and (*μ*^′^,*σ*,*m*) [13] summarized in Appendix Appendix A: peak descriptors and parameters.

We also make use of the following empirically observable properties of peaks in real IMSCs that concern the peak widths on both the IRM axis and the retention time axis. The width can be described as the length *ω*_1/2_ of the interval around the mode where the peak height is at least half of its maximum height. For a (symmetric) Gaussian distribution, there is a linear relation between the standard deviation *σ* and *ω*_1/2_:

(2) $$ \omega_{\nicefrac{1}{2}} = \phi \cdot \sigma \qquad \text{with}\, \, \phi = 2 \sqrt{2 \ln 2} \approx 2.3548 \,.   $$

This relation approximately holds as well for not too skewed Inverse Gaussian distributions and is a good approximation to estimate its descriptor *σ* approximately from an empirically observed *ω*_1/2_.

Given the mode *d*^∗^ of a peak in drift time (in ms), we can estimate its descriptors (*m*,*σ*,*μ*^′^) in IRM units as follows. Recall that the IRM mode (in V s cm ^−2^) is simply *m*=*C*_t|d_·*d*^∗^, where *C*_t|d_ is the conversion constant between drift time and IRM (see Section ‘Data from MCC/IMS measurements’). Spangler *et al.* [14] empirically derived that $\omega _{\nicefrac {1}{2}} = \sqrt { (11.09\, \mathcal {D} \, d^{*}) / v_{\text {d}}^{2} + d_{\text {grid}}^{2} }$, where  is the diffusion coefficient, *v*_d_ the drift velocity. Using the Einstein relation [15],  can be computed as $\mathcal {D} = k \mathcal {K_{\text {B}}}\mathcal {T} / q$, where *k* is the ion mobility, $\mathcal {K_{\text {B}}}$ the Boltzmann constant,  the absolute temperature and *q* the electric charge. We then use (2) to estimate *σ*≈*ω*_1/2_/*ϕ*. Finally, the mean is empirically found to be $\mu ' \approx C_{\text {t}|\text {d}} \cdot \left (d^{*} + \sqrt {(4.246 \cdot 10^{-5})^{2} + (d^{*})^{2} / 585048.1633} \right)$.

On the retention time axis, the peak width *ω*_1/2_ grows approximately linearly with retention time, i.e., there are constants r_width_offset>0 and r_width_factor>0 such that width of a peak with maximum at retention time *r* is approximately

(3) $$ \xi(r) := r \cdot \texttt{r\_width\_factor} + \texttt{r\_width\_offset} \,.   $$

### Optimization methods

The online peak extraction algorithm makes use of non-linear unconstrained minimization, similar to non-linear least squares, and of the EM algorithm. Both methods are summarized here.

#### Non-linear Least Squares

The NLLS method is an iterative method to estimate parameters *θ*=(*θ*_1_,…,*θ*_*q*_) of a supposed parametric function *f*, given *n* observed data points (*x*_1_,*y*_1_),…,(*x*_*n*_,*y*_*n*_) with *y*_*i*_=*f*(*x*_*i*_;*θ*). The idea is to minimize the quadratic error $\sum _{i=1}^{n}\, {r^{2}_{i}}(\theta)$ between the function and the observed data, where *r*_*i*_(*θ*):=*y*_*i*_−*f*(*x*_*i*_;*θ*) is the *residual* of the *i*-the datapoint. The necessary optimality condition is $\sum _{i}\, r_{i}(\theta) \cdot \partial r_{i}(\theta) / \partial \theta _{j} = 0$ for all *j*. If *f* is linear in *θ* (e.g., a polynomial in *x* with *θ* being the polynomial coefficients, a setting called polynomial regression), then the optimality condition results in a linear system, which can be solved in closed form. However, often *f* is not linear in *θ* and we obtain a non-linear system, which is solved iteratively, given initial parameter values, by linearizing it in each iteration. Details and different algorithms for NLLS can be found in the literature ([16], Chapter 10). In this paper, we use a different, non-symmetric loss function, but apply similar techniques to solve the problem (see below).

#### The EM algorithm for mixtures with heterogeneous components

The observed data *x*=(*x*_1_,…,*x*_*n*_) is viewed as a *sample* from a *mixture* of probability distributions, where the mixture density is specified by $f(x_{i} \,|\, \omega, \theta) = \sum _{c=1}^{C}\, \omega _{c}\; f_{c}(x_{i} \,|\, \theta _{c})$. Here *c* indexes the *C* different component distributions *f*_*c*_, where *θ*_*c*_ denotes the parameters of *f*_*c*_, and *θ*=(*θ*_1_,…,*θ*_*C*_) is the collection of all parameters. The mixture coefficients satisfy *ω*_*c*_≥0 for all *c*, and $\sum _{c}\, \omega _{c} = 1$. Unlike in most applications, where all component distributions *f*_*c*_ are multivariate Gaussians, here the *f*_*c*_ are of different types (e.g., uniform and Inverse Gaussian). The goal is to determine the parameters *ω* and *θ* such that the probability of the observed sample is maximal (maximum likelihood paradigm). Since the resulting optimization problem is non-convex in (*ω*,*θ*), the EM algorithm is an iterative method that will converge to a local optimum [17] in parameter space. The EM algorithm consists of two repeated steps: The E-step (expectation) estimates the expected membership of each data point in each component and then the component weights *ω*, given the current model parameters *θ*. The M-step (maximization) estimates maximum likelihood parameters *θ*_*c*_ for each parametric component *f*_*c*_ individually, using the expected memberships as hidden variables that decouple the model.

##### E-Step.

To estimate the expected membership *W*_*i*,*c*_ of data point *x*_*i*_ in each component *c*, the component’s relative probability at that data point is computed, such that $\sum _{c}\, W_{i,c} = 1$ for all *i*. Then the new component weight estimates $\omega ^{+}_{c}$ are the averages of *W*_*i*,*c*_ across all *n* data points.

(4) $$ W_{i,c} = \frac{\omega_{c}\, f_{c}(x_{i} \,|\, \theta_{c})}{\sum_{k}\, \omega_{k}\, f_{k}(x_{i} \,|\, \theta_{k})}, \qquad \omega^{+}_{c} = \frac{1}{n} \sum_{i=1}^{n}\, W_{i,c},   $$

##### Convergence.

After each M-step of an EM cycle, we compare *θ*_*c*,*q*_ (old parameter value) and $\theta ^{+}_{c,q}$ (updated parameter value), where *q* indexes the elements of *θ*_*c*_, the parameters of component *c*. We say that the algorithm has converged when the relative change $\kappa _{c,q} := |\theta _{c,q}^{+} - \theta _{c,q}| \,/\, \max \left (|\theta _{c,q}^{+}|,|\theta _{c,q}| \right)$ drops below a given threshold thresh for all *c*,*q*, (if $\theta _{c,q}^{+} = \theta _{c,q} = 0$, we set *κ*_*c*,*q*_:=0).

Having reviewed the necessary background, we now describe the methods we use for peak extraction from IMSCs.

## Denoising and baseline correction

### Background

A major challenge during peak detection in an IM spectrum is to find peaks that only slightly exceed the background noise level in a spectrum *S*=(*S*_*t*_). To determine whether the intensity *S*_*t*_ at coordinate *t* belongs to a peak region or can be solely explained by background noise, we propose a method based on the EM algorithm. It runs in $\mathcal {O}(\tau |T|)$ time where *τ* is the number of EM iterations.

### Mixture model

Based on observations of IM spectra signal intensities, we assume that

- the noise intensity has a Gaussian distribution over low intensity values with mean *μ*_N_ and standard deviation *σ*_N_,
  $$p_{\text{N}}(s \,|\, \mu_{\text{N}}, \sigma_{\text{N}}) = \frac{1}{\sqrt{2 \pi} \, \sigma_{\text{N}}} \cdot \exp \big(-(s - \mu_{\text{N}})^{2} / (2\, \sigma_{\text{N}}^{2}) \big) $$
- the true signal intensity has an Inverse Gaussian distribution with mean *μ*_S_ and shape parameter *λ*_S_, i.e.,
  $$p_{\text{S}}(s \,|\, \mu_{\text{S}}, \lambda_{\text{S}}) \,=\, \sqrt{\lambda_{\text{S}} / (2 \pi s^{3})} \cdot \exp \big(-\lambda_{\text{S}} (s - \mu_{\text{S}})^{2} / (2 \mu_{\text{S}}^{2} s) \big) $$
- there is an unspecific background component which is not well captured by either of the two previous distributions; we model it by the uniform distribution over all intensities,
  $$p_{\text{B}}(s) = 1 / (\max(S) - \min(S)), $$
  and we expect the weight *ω*_B_ of this component to be close to zero in standard IM spectra. High weights indicate an anomaly during the measurement.

**Figure 3:**
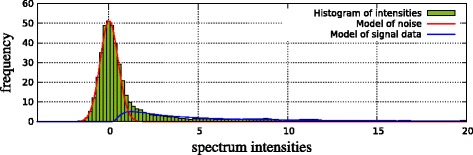
Histogram of an arbitrary IM spectrum (green bars) and estimated distribution of the noise component (red line) and of the signal component (blue line). Parameters for both components were estimated with the EM algorithm.

We interpret the observed spectrum *S* as a sample of a mixture of these three components with unknown mixture coefficients. To illustrate this approach, consider Figure 3, which shows the empirical intensity distribution (histogram) of an arbitrary spectrum, together with the estimated components (except the uniform distribution, which has the expected coefficient of almost zero).

It follows that there are six independent parameters to estimate: *μ*_N_, *σ*_N_, *μ*_S_, *λ*_S_ and weights *ω*_N_,*ω*_S_,*ω*_B_ (noise, signal, background, where *ω*_B_=1−*ω*_N_−*ω*_S_).

### Initial parameter values

Background noise intensities are assumed to follow a Gaussian distribution at small intensity values. We can determine its approximate mean *μ*_N_ and standard deviation *σ*_N_ by considering the first and last 10*%* of data points in each spectrum.

The initial weight of the noise component is set to cover most points covered by this Gaussian distribution, i.e., *ω*_N_:=|{*t*∈*T* | *S*_*t*_≤*μ*_N_+3 *σ*_N_}| / |*T*|.

We assume that almost all of the remaining weight belongs to the signal component, thus *ω*_S_=(1−*ω*_N_)·0.999, and *ω*_B_=(1−*ω*_N_)·0.001.

To obtain initial parameters for the signal model, let *I*^′^:={*t*∈*T* | *S*_*t*_>*μ*_N_+3 *σ*_N_} (the complement of the intensities that are initially assigned to the noise component). We set $\mu _{\text {S}} = \left (\sum _{t \in I'}\, (S_{t} - \mu _{\text {N}})\right) / |I'|$ and $\lambda _{\text {S}} = (\sum _{t \in I'}\, (1/(S_{t} - \mu _{\text {N}}) - 1 / \mu _{\text {S}}))^{-1}$ (which are the maximum likelihood estimators for Inverse Gaussian parameters).

### E-step

The hidden parameters *W*_*t*,*c*_ are computed using (4), where the three component distributions *f*_*c*_ are the three component densities *p*_N_,*p*_S_,*p*_B_ with their parameters and the data *x* is a mean-smoothed version of the original spectrum *S*: $x_{t} := \frac {1}{2\alpha +1} \cdot \sum _{t' = t - \alpha }^{t + \alpha } S_{t'}$, where the smoothing window margin is *α*:=(1/2)·*d*_grid_·*C*_t|d_·|*T*|/*T*_last_. (Here *d*_grid_ is the grid opening time of the spectrometer and *T*_last_ is the maximum IRM in *T*).

### Maximum likelihood estimators

In the maximization step (M-step) we estimate maximum likelihood parameters for the non-uniform components using the original intensities of *S* again.

(5) $$\begin{array}{*{20}l} \mu_{\text{N}} &= \frac{\sum_{t}\, W_{t, \text{N}} \cdot S_{t}}{\sum_{t}\, W_{t, \text{N}}},  \end{array} $$

(6) $$\begin{array}{*{20}l} \mu_{\text{S}} &= \frac{\sum_{t}\, W_{t, \text{S}} \cdot (S_{t} - \mu_{\text{N}})}{\sum_{t}\, W_{t, \text{S}}},  \end{array} $$

(7) $$\begin{array}{*{20}l} \sigma^{2}_{\text{N}} &= \frac{\sum_{t}\, W_{t, \text{N}} \cdot (S_{t} - \mu_{\text{N}})^{2} }{ \sum_{t}\, W_{t, \text{N}} },  \end{array} $$

(8) $$\begin{array}{*{20}l} \lambda_{\text{S}} &= \frac{\sum_{t}\, W_{t, \text{S}} }{ \sum_{t}\, W_{t, \text{S}} \cdot (1 / (S_{t} - \mu_{\text{N}}) - 1 / \mu_{\text{S}})}  \end{array} $$

for all *t*∈*T*.

### Final step

After convergence, we correct the baseline and remove noise: We first subtract *μ*_N_ from the signal value and then reduce the remaining value by the estimated noise weight. The corrected spectrum *S*^+^ is

$$S^{+}_{t} := \max \big\{ (1 - W_{t,\text{N}}) (S_{t} - \mu_{\text{N}}), 0 \big\}, \quad t \in T. $$

## Reducing a spectrum to peak models

### Background

The idea of processing a single (noise-reduced) IM spectrum *S* is to deconvolute it into separate components described with statistical distribution functions. Several components appear in each spectrum besides the peaks, namely the previously described RIP and the tailing described in Section ‘Data from MCC/IMS measurements’. and background noise. We first determine and remove the RIP tailing function and then determine the peak parameters (including the RIP).

### Determining the tailing function

The tailing function appears as a baseline in every spectrum (see Figure 2 for an example). Its shape and scale changes from spectrum to spectrum; so it has to be determined in each spectrum and subtracted in order to extract peaks from the remaining signal in the next step. Empirically, we observe that the tailing function *f*(*t*) can be described by a scaled shifted Inverse Gaussian, *f*(*t*)=*v*·*g*(*t*;*μ*,*λ*,*o*) with *g* given by (1). The goal is to determine the parameters *θ*=(*v*,*μ*,*λ*,*o*) such that *f*_*θ*_(*t*) under-fits the given data *S*=(*S*_*t*_), as shown in Figure 2.

Let *r*_*θ*_(*t*):=*S*(*t*)−*f*_*θ*_(*t*) be the residual function for a given choice *θ* of parameters. As we want to penalize *r*(*t*)<0 but not (severely) *r*(*t*)>0, we use the following non-symmetric loss function that depends on a threshold parameter *γ*>0:

$$e_{t}(\theta; \gamma) := \left\{ \begin{array}{lr} r_{\theta}(t)^{2} / 2 &\text{if}\,\, r_{\theta}(t)<\gamma \,, \\ \gamma \cdot r_{\theta}(t) - \gamma^{2} / 2 &\text{if}\,\, r_{\theta}(t)\geq \gamma \,. \end{array} \right. $$

That is, the loss at time *t* is the residual squared when it has a negative or small positive value less than the given threshold *γ*>0, but becomes a linear function of the residual for larger positive residuals.

The goal is to find *θ* to minimize the total loss $L(\theta) := \sum _{t}\, e_{t}(\theta ; \gamma)$ for the given spectrum *S* and given *γ*>0. We use gradient descent to solve this nonlinear optimization problem, to which we refer as non-linear loss minimization (NLLM).

To estimate the tailing function,

1. we determine reasonable initial values for the parameters *θ*=(*v*,*μ*,*λ*,*o*) (see below),
2. we solve NLLM with $\gamma = \sigma _{\text {N}}^{2}$ to estimate the scaling factor *v*, leaving the other parameters fixed,
3. we solve NLLM with $\gamma = \sigma _{\text {N}}^{2}$ to estimate all four parameters,
4. we solve NLLM with $\gamma = \sigma _{\text {N}}^{2}/100$ to re-estimate the scaling factor *v*

where *σ*_N_ is the standard deviation of the noise as described in Section ‘Denoising and baseline correction’.

The initial parameter values (*v*,*μ*,*λ*,*o*) are determined as follows: For the scaling factor, we initially set $v = (1/2) \sum _{t \leqslant |T|}\, S_{t}$. For the other parameters, we first estimate the descriptors (*μ*^′^,*σ*,*m*) as described below and then use the correspondence to the parameters listed in Appendix Appendix A: peak descriptors and parameters. The initial *σ* is set to the standard deviation of the whole RIP-only spectrum. We determine the initial *m* as the RIP mode. It is a property of the Inverse Gaussian distributions under consideration such that the mean *μ*^′^ can only range within the interval *I*= [*m*,*m*+0.7*σ*]. To obtain an appropriate value for *μ*^′^, an auxiliary offset variable *o*^′^ is set to the largest IRM left of the RIP mode where the signal is below *σ*_N_, and *μ*^′^ it is increased in small steps within *I*. The candidate descriptors (*μ*^′^,*σ*,*m*) are converted into corresponding parameters (*μ*,*λ*,*o*) until *o*≥*o*^′^. The so obtained parameters constitute the initial parameter values.

### Extracting peak parameters from a single spectrum

To extract all peaks from a spectrum (from left to right), we repeat three sub-steps:

1. scanning for a potential peak, starting where the previous iteration stopped;
2. determining peak parameters (Inverse Gaussian distribution);
3. subtracting the peak from the spectrum and continuing with the remainder.

**Scanning.** The algorithm scans for peaks, starting at the left end of *S*, by sliding a window of a given width across *S* and fitting a quadratic polynomial model to the data points within the window. The window width (in index units) is related to the grid opening time *d*_grid_ of the spectrometer and given as *d*_grid_/*D*_last_·|*D*| data points, where *D*_last_ is the maximum (last) drift time measured.

The model is built in drift time (not IRM). Let *f*(*d*;*θ*)=*θ*_2_*d*^2^+*θ*_1_*d*+*θ*_0_ be the fitted quadratic polynomial inside the window. We call a window a *peak window* if the following conditions are fulfilled:

- the extreme drift time *d*^∗^=*θ*_1_/(2*θ*_2_) lies within the drift times of the window;
- the extreme drift time *d*^∗^ indicates a maximum (i.e., *θ*_2_<0);
- the maximum is sufficiently high above the noise level (which is zero after preprocessing): *f*(*d*^∗^;*θ*)≥*σ*_N_

The first condition can be more strongly restricted to achieve more reliable results, by shrinking the interval towards the center of the window. If no peak is found, the moving window is shifted one index forward. If a peak is detected, the window is shifted half the window length forward before the next scan begins, but first the peak parameters are computed.

**Determining peak parameters.** As described in Section ‘Peak models’, we can estimate all peak descriptors (*m*,*σ*,*μ*^′^) from its mode *d*^∗^ in drift time. We convert them into the parameters (*μ*,*λ*,*o*) of the Inverse Gaussian parameterization (see Appendix Appendix A: peak descriptors and parameters). The scaling factor *v* for the peak is *v*=*f*(*d*^∗^;*θ*)/*g*(*m*;*μ*,*λ*,*o*).

The model function is subtracted from the spectrum, and the next iteration is started with a window shifted by *α* index units (consider Section ‘E-step’). For each spectrum, the output of this step is a *spectrum peak list*, which is a set of parameters for a mixture of weighted Inverse Gaussian models describing the peaks.

## Aligning consecutive spectrum peak lists

### Background

Having a set of peak parameters for each spectrum, the question arises how to merge the sets *P*=(*P*_*i*_) and $P^{+}=(P^{+}_{j})$ of two consecutive spectra. For each peak *P*_*i*_, we have stored the Inverse Gaussian parameters *μ*_*i*_,*λ*_*i*_,*o*_*i*_, the peak descriptors *μi*′,*σ*_*i*_,*m*_*i*_ (mean, standard deviation, mode) and the scaling factor *v*_*i*_, and similarly so for the peaks $P^{+}_{j}$. The idea is to compute a global alignment similar to the Needleman-Wunsch method [18] between *P* and *P*^+^. We need to specify how to score aligning *P*_*i*_ to $P^{+}_{j}$ and how to score leaving a peak unaligned (i.e., a gap).

### Scoring peak alignments

The score *Z*_*ij*_ for aligning *P*_*i*_ to $P^{+}_{j}$ is chosen by evaluating *P*_*i*_’s density function at the new mode $m_{j}^{+}$ and comparing it to “typical” value an approximate standard deviation away from the mode (at *m*_*i*_+*δ*, where *δ*:=*d*_grid_·*C*_t|d_/*ϕ*), resulting in the log-odds score

$$ \zeta_{i,j} = \ln \left(\frac{g(m_{j}^{+};\, \mu_{i}, \lambda_{i}, o_{i}) }{ g(m_{i} + \delta;\, \mu_{i}, \lambda_{i}, o_{i})} \right). $$

Alternatively, leaving a peak unmatched results in a gap score of zero.

Applying Needleman-Wunsch global alignment, we can compute the optimal score of aligning the first *i* peaks in the former spectrum with the first *j* peaks in the current spectrum by dynamic programming. We initialize a matrix *Z*, setting all *Z*_*i*,0_ and *Z*_0,*j*_ to zero and then compute, for *i*≥1 and *j*≥1,

$$Z_{i,j} = \max \left\{ \begin{array}{lr} Z_{i-1,j-1} + \zeta_{i,j},\\ Z_{i-1,j}, \\ Z_{i,j-1}. \end{array} \right. $$

### Obtaining peak chains

The alignment is obtained with a traceback, recording the optimal case in each cell, as usual. There are three cases to consider.

- If $P^{+}_{j}$ is not aligned with a peak in *P*, potentially a new peak starts at this retention time. Thus model $P^{+}_{j}$ is put into a new peak chain.
- If $P^{+}_{j}$ is aligned with a peak *P*_*i*_, the chain containing *P*_*i*_ is extended with $P^{+}_{j}$.
- All peaks *P*_*i*_ that are not aligned to any peak in *P*^+^ indicate the end of a peak chain at the current retention time.

All completed peak chains are forwarded to the next step, two-dimensional peak model estimation.

## Estimating 2-D peak models

### Background

Let *C*=(*P*_1_,…,*P*_*n*_) be a chain of one-dimensional Inverse Gaussian models. The goal of this step is to estimate a two-dimensional peak model (product of two one-dimensional Inverse Gaussians) from the chain, as described in Section ‘Peak models’, or to reject the chain if the chain does not fit such a model well. Potential problems are that a peak chain may contain noisy 1-D peaks truncated at their borders, consist only of noise or in fact consist of several consecutive 2-D peaks at the same drift time and successive retention times.

### Estimating the parameters

As discussed in Section ‘Peak models’, the half-height width *ω*_1/2_ in retention time of a peak centered at retention time *r* can be described by an affine function *ξ*(*r*), Eq. (3), and *ω*_1/2_ can be converted to the corresponding number of data points (window width).

We have the parameters $({\hat {v}}_{i}, {\hat {\mu }}_{i, \text {t}}, {\hat {\lambda }}_{i, \text {t}}, {\hat {o}}_{i, \text {t}})$ for each individual peak *i*=1,…,*n* in a peak chain, and the corresponding descriptors $({\hat {\mu }}'_{i,\text {t}}, {\hat {\sigma }}_{i,\text {t}}, {\hat {m}}_{i,\text {t}})$, as well as the associated retention time *r*_*i*_ and peak height $h_{i} = {\hat {v}}_{i} \cdot g({\hat {m}}_{i,\text {t}}; {\hat {\mu }}_{i, \text {t}}, {\hat {\lambda }}_{i, \text {t}}, {\hat {o}}_{i, \text {t}})$.

We proceed similarly to Section ‘Extracting peak parameters from a single spectrum’ by fitting quadratic polynomials *b*(*r*;*θ*)=*θ*_2_*r*^2^+*θ*_1_*r*+*θ*_0_ in sliding windows of the appropriate width *ξ*(*r*_*i*_) such that *h*_*i*_≈*b*(*r*_*i*_;*θ*).

Having found a window that fits a peak, we estimate initial descriptors for an Inverse Gaussian model in retention time as follows:

$$\begin{array}{*{20}l} v_{\text{r}}' &= -{\theta_{1}^{2}} / (4\theta_{2}) + \theta_{0}, \\ \sigma_{\text{r}} &= \sqrt{v_{\text{r}}' / (2|\theta_{2}|)}, \\ m_{\text{r}} &= -\theta_{1} / (2\theta_{2}), \\ \mu'_{\text{r}} &= m_{\text{r}} + \xi(m_{\text{r}}) \,/\, (4\,\phi). \end{array} $$

The descriptors are then converted into model parameters (see Appendix Appendix A: peak descriptors and parameters).

After processing each window, we have obtained a list of size, say, *k*, of Inverse Gaussian distributions. We expect these distributions to be a mixture of *k* overlapping peaks in a single peak chain. To obtain an optimal deconvolution, we first normalize the volume factors *v*r′ of the *k* components to obtain *v*_r,*j*_ such that $\sum _{j=1}^{k}\, v_{\text {r},r} = 1$ and then apply the EM algorithm. As a byproduct, we obtain an (*n*×*k*) matrix *M*=(*M*_*i*,*j*_) that determines the membership probability for each of the *n* data points (*r*_*i*_,*h*_*i*_) to each of the *k* models.

To obtain the Inverse Gaussian distribution parameters in the IRM dimension for each of the *k* models, we first compute model descriptors using a membership-weighted average over the individual model descriptors: For *j*∈{1,…,*k*}, let

$$\begin{array}{*{20}l} \overline{M}_{j} &:= \sum_{i \leqslant n}\, M_{i, j}, \\ \mu'_{j,\text{t}} &:= \frac{1}{\overline{M}_{j}} \sum_{i \leqslant n}\, M_{i, j} \cdot {\hat{\mu}}'_{i,\text{t}}, \\ \sigma_{j,\text{t}} &:= \frac{1}{\overline{M}_{j}} \sum_{i \leqslant n}\, M_{i, j} \cdot {\hat{\sigma}}_{i,\text{t}}, \\ m_{j,\text{t}} &:= \frac{1}{\overline{M}_{j}} \sum_{i \leqslant n}\, M_{i, j} \cdot {\hat{m}}_{i,\text{t}}. \end{array} $$

We then convert these descriptors back into model parameters (Appendix Appendix A: peak descriptors and parameters). The final peak volume is computed as $v_{j}^{*} = v_{j, \text {r}}' \cdot \sum _{i \leqslant n} v_{i, \text {t}}$.

For every model *j*∈{1,…,*k*}, we check the following conditions:

- The width at half height in the retention time dimension has approximately the expected size (cf. Eqs. (2), (3)): *ξ*(*m*_*j*,r_)/2≤*σ*_*j*,r_/*ϕ*<2·*ξ*(*m*_*j*,r_),
- The peak height at its maximum is sufficiently above the noise level: $v_{j}^{*} \cdot g(m_{j, \text {t}}; \mu _{j, \text {t}}, \lambda _{j, \text {t}}, o_{j, \text {t}}) \cdot g(m_{j, \text {r}}; \mu _{j, \text {r}}, \lambda _{j, \text {r}}, o_{j, \text {r}}) \geq \texttt {noise\_margin} \cdot \sigma _{_{\text {N}}}$, where noise_margin>0 is a tunable parameter,
- the Inverse Gaussian peak model *g* in retention time correlates well (in terms of the Pearson product-moment correlation coefficient *ρ*) with its quadratic approximation *b* in a window around the mode. More precisely, consider the window *W*= [*m*_*j*,r_−*ξ*(*m*_*j*,r_)/*ϕ*, *m*_*j*,r_+*ξ*(*m*_*j*,r_)/*ϕ*], the model vector *G*=*g*(*x*;*μ*_*j*,r_,*λ*_*j*,r_,*o*_*j*,r_) for *x*∈*W* and the quadratic approximation vector *B*=*b*_*j*_(*x*;*θ*) for *x*∈*W*, and test whether the Pearson correlation satisfies *ρ*(*G*,*B*)≥*ρ*_min_.

If all conditions are satisfied, we have identified a 2-D peak model $(v_{j}^{*}, \mu _{j, \text {t}}, \lambda _{j, \text {t}}, o_{j, \text {t}}, \mu _{j, \text {r}}, \lambda _{j, \text {r}}, o_{j, \text {r}})$. Otherwise the model is discarded.

## Peak clustering

### Background

We now consider a series of IMS measurements, for each of which we have extracted peaks available in the form of parameter vectors or descriptors. The question arises how to decide which descriptors in different measurements represent the same peak (and hence potentially the same VOC).

Let *X* be the union of peak locations in all measurements, let |*X*|=:*n*, and let *X*_*i*,R_ be the retention time of peak *i* and *X*_*i*,T_ its IRM. We introduce a clustering approach using the EM algorithm with two-dimensional Gaussian mixtures that differs from the standard approach by its ability to dynamically adjust the number of clusters in the process.

### Mixture model

We assume that the measured retention times and IRMs belonging to peaks from the same compound are independently normally distributed in both dimensions around the (unknown) true retention time and IRM. Let *θ*_*j*_:=(*μ*_*j*,R_,*σ*_*j*,R_,*μ*_*j*,T_,*σ*_*j*,T_) be the parameters for component *j*, and let *f*_*j*_(*x*^′^*givenθ*_*j*_ be a two-dimensional Gaussian product distribution for a peak location *x*=(*x*_R_,*x*_T_) with these parameters.

The mixture distribution is $f(x) = \sum _{j=1}^{C}\, \omega _{j}\, f_{j}(x\,|\, \theta _{j})$ with a yet undetermined number *C* of clusters. Note that there is no “background” model component.

### Initial parameter values

In the beginning, we initialize the algorithm with as many clusters as peaks, i.e., we set *C*:=*n*. This assignment makes a background model obsolete, because all peaks are assigned to at least one cluster. All clusters get as start parameters for *μ*_*j*,R_,*μ*_*j*,T_ the original retention time and IRM of peak location *X*_*j*_, respectively, for *j*=1,…,*n*. We set *σ*_*j*,T_:=t_width>0 and *σ*_*j*,R_:=*ξ*(*X*_*j*,R_)/*ϕ*.

### Dynamic adjustment of the number of clusters

After computing weights in the E-step, but before starting the M-step, we dynamically adjust the number of clusters by merging clusters whose centers are close to each other. Every pair *j*<*k* of clusters is compared in a nested for-loop. When |*μ*_*j*,T_−*μ*_*k*,T_|<t_width and |*μ*_*j*,R_−*μ*_*k*,R_|<*ξ*(max{*μ*_*j*,R_,*μ*_*k*,R_}), then clusters *j* and *k* are merged by summing the EM weights: *ω*^+^:=*ω*_*j*_+*ω*_*k*_ and *W*_*i*,+_:=*W*_*i*,*j*_+*W*_*i*,*k*_ for all *i*. The summed weights are assigned to the location of the cluster with larger weight. (The re-computation of the parameters happens immediately after merging in the maximization step). The comparison order may matter in rare cases for deciding which peaks are merged first, but since new means and variances are computed, possible merges that were omitted in the current iteration will be performed in the next iteration.

This merging step is applied after in the second EM iteration, since the cluster means need at least one iteration to move towards each other.

### Maximum likelihood estimators

The maximum likelihood estimators for mean and variance of a two-dimensional Gaussian are the standard ones, taking into account the membership weights,

(9) $$\begin{array}{*{20}l} \mu_{j,d} &= \frac{\sum_{i=1}^{n}\, W_{i, j} \cdot X_{i, d}}{\sum_{i=1}^{n}\, W_{i, j}}, & d\in\{\text{T,R}\}, \end{array} $$

(10) $$\begin{array}{*{20}l} \sigma_{j,d}^{2} &= \frac{\sum_{i=1}^{n}\, W_{i, j} \cdot (X_{i, d} - \mu_{j, d})^{2}} {\sum_{i=1}^{n}\, W_{i, j}}, & d\in\{\text{T,R}\}, \end{array} $$

for all components *j*=1,…,*C*.

One problem using this approach emerges from the fact that initially each cluster contains only one peak, leading to an estimated variance of zero in many cases. To prevent this, minimum values are enforced such that *σ*_*j*,T_≥t_width and *σ*_*j*,R_≥*ξ*(*μ*_*j*,R_)/*ϕ* for all *j*.

### Final step

The EM loop terminates when no merging occurs and the convergence criteria for all parameters are fulfilled. The resulting membership weights determine the number of clusters as well as the membership coefficient of peak location *X*_*i*_ to cluster *j*. If a hard clustering is desired, the merging step has to be traced.

## Evaluation

We evaluate different properties of the online method:

1. the quality of reducing a single spectrum to a peak list (denoising/baseline correction (Section ‘Denoising and baseline correction’) and spectrum reduction (Section ‘Reducing a spectrum to peak models’),
2. the execution time of both steps,
3. the quality of the new clustering approach,
4. the correlation between manual annotations on full IMSCs by a computer-assisted expert and our automated online extraction method.

**Parameters.** For evaluation measurements, the MCC was adjusted to a temperature of 40°C and throughput of 150 mL min ^−1^. The IMS had a voltage of 4380 V, a grid opening time of 300 *μ*s and a throughput of 150 mL min ^−1^. We chose the following parameters [9]:

- r_width_offset = 2.5 s (width offset for peaks in retention time),
- r_width_factor = 0.06 (width slope for peaks in retention time),
- t_width = 0.003 V s cm ^−2^ (standard deviation for peaks in IRM),
- thresh (convergence threshold; value varies within evaluation),
- noise_margin = 4 (factor multiplied with standard deviation of background noise for minimal peak height),
- *ρ*_min_ = 0.95 (minimal Pearson product-moment correlation coefficient).

### Quality of single spectrum reduction

In a first experiment, we tested the quality of the spectrum reduction method using an idea by Munteanu and Wornowizki [19] that determines the agreement between an observed set of data points, interpreted as an empirical distribution function *F* (the data) and a model distribution *G* (the mixture distribution obtained from the peak list parameters). The approach writes $F = \tilde {s} \cdot G + (1 - \tilde {s}) \cdot H$ with $\tilde {s} \in \,[0,1]$, where *H* is a non-parametric distribution whose inclusion ensures the fit of the model *G* to the data *F*. If the weight $\tilde {s}$ is close to 1.0, then *F* is a plausible sample from *G*. We compare the original spectra and reduced spectra (peaks from peak lists) from a previously used dataset [20]. This set contains 69 measurements preprocessed with a 5×5 average. Every measurement contains 1200 spectra. For each spectrum in all measurements, we computed the reduced spectrum model and determined $\tilde {s}$. Over 92*%* of all 82 000 models achieved $\tilde {s} = 1$ and over 99*%* reached $\tilde {s} \ge 0.9$. No $\tilde {s}$ dropped below 85*%*. In summary, spectrum reduction provides an accurate parametric representation of most spectra.

### Execution time

We tested our method on two different platforms, (1) a desktop PC with Intel(R) Core(TM) i5 2.80GHz CPU, 8GB memory, Ubuntu 12.04 (64bit) OS and (2) a Raspberry Pi [21] type B with ARM1176JZF-S 700MHz CPU, 512 MB memory, Raspbian Wheezy (32bit) OS, once with the factory defaults of 700 MHz and once overclocked up to 900 MHz. The Raspberry Pi was chosen because it is a complete credit-card-sized low-cost single-board computer with low CPU and power consumption (3.5 w). This kind of device is appropriate for data analysis in future mobile measurement devices.

Recall that each spectrum contains 12 500 data points. It is current practice to analyze not the full spectra, but aggregated ones, where five consecutive values are averaged. Here we consider the full spectra, slightly aggregated ones (average over two values, 6 250 data points) and standard aggregated ones (average over five values, 2 500 data points). We measured the average execution time of denoising, baseline correction and consecutive spectrum reduction. Table 1 shows the results. It is remarkable that at the highest resolution (Average 1) the Raspberry Pi with 900 MHz keeps barely the time bound of 100 ms between consecutive spectra. At lower resolutions, the Raspberry Pi satisfies the time restrictions easily. The desktop PC copes with the analysis effortless on any setting.

**Table 1:** **Average processing time of denoising, baseline correction and spectrum reduction on two platforms with different clock rates, averaging methods (single spectra, averages of 2 and 5 spectra) and convergence thresholds** thresh

thresh	***Platform***	***Avg 1***	***Avg 2***	***Avg 5***
0.1*%*	Desktop PC	4.36 ms	2.09 ms	0.88 ms
	Rasp. Pi (700 MHz)	119.48 ms	55.02 ms	21.82 ms
	Rasp. Pi (900 MHz)	97.19 ms	43.62 ms	17.42 ms
1.0*%*	Desktop PC	4.26 ms	2.01 ms	0.66 ms
	Rasp. Pi (700 MHz)	116.69 ms	52.63 ms	16.99 ms
	Rasp. Pi (900 MHz)	94.03 ms	41.46 ms	13.48 ms

We found that in the steps that use the EM algorithm, on average 25–30 EM iterations were necessary for a precision of thresh:=0.001 (i.e., 0.1*%*) (see Convergence in Section ‘The EM algorithm for mixtures with heterogeneous components’). Relaxing the threshold from 0.001 to 0.01 halved the number of iterations without noticeable difference in the resulting estimated parameters.

### Clustering

To evaluate peak clustering methods, we simulate peak locations according to locations in real MCC/IMS datasets, together with the true partition  of peaks.

Most of the detected peaks appear in a small dense area early in the measurement. The remaining peaks are distributed widely, which is referred to as the sparse area (we let the areas overlap such that the dense are is contained in the sparse area). The areas approximately have the following boundaries (in units of (V s cm ^−2^, s) from lower left to upper right point, cf. Figure 1:

- measurement: (0,0),(1.45,600)
- dense area: (0.5,4),(0.7,60)
- sparse area: (0.5,4),(1.2,450)

Peak clusters are ellipsoidal and dense. From [9], we know the minimum required distance between two peaks in order to be identified as two separate compounds. We simulate 30 peak cluster centroids in the dense area and 20 in the sparse area, all picked randomly and uniformly distributed in the respective area. We then randomly pick the number of peaks per cluster. We also randomly pick the distribution of peaks within a cluster. Since we do not know the actual distribution model, we decided to simulate with three models: normal (n), exponential (e) and uniform (u) distribution with the following densities:

$$ \begin{aligned} & f_{\text{n}}(r, t \,|\, \mu_{\text{t}}, \sigma_{\text{t}}, \mu_{\text{r}}, \sigma_{\text{r}}) \\ &\quad = \mathcal{N}(t \,|\, \mu_{\text{t}}, \sigma_{\text{t}}) \cdot \mathcal{N}(r \,|\, \mu_{\text{r}}, \sigma_{\text{r}}) \\ & f_{\text{e}}(r, t \,|\, \mu_{\text{t}}, \lambda_{\text{t}}, \mu_{\text{r}}, \lambda_{\text{r}}) \\ &\quad = \lambda_{\text{t}} \lambda_{\text{r}} \exp \big(- (\lambda_{\text{t}} |t-\mu_{\text{t}}| + \lambda_{r} |r-\mu_{\text{r}}|) \big) / 4 \\ & f_{\text{u}}(r, t \,|\, \mu_{\text{t}}, \nu_{\text{t}}, \mu_{\text{r}}, \nu_{\text{r}}) \\ &\quad = \left\{ \begin{array}{lr} (\pi \nu_{\text{t}} \nu_{\text{r}})^{-1} & \text{if} \frac{|t-\mu_{\text{t}}|^{2}}{\nu_{\text{t}}^{2}} + \frac{|r-\mu_{\text{r}}|^{2}}{\nu_{\text{r}}^{2}} \leqslant 1 \\ 0 & \text{otherwise} \end{array} \right. \end{aligned} $$

Here (*μ*_t_,*μ*_r_) is the coordinate of the centroid with RIM in Vs/cm^2^ and retention time in s. For the normal distribution, we used *σ*_t_=0.002 and *σ*_r_=*μ*_r_·0.002+0.2. For the exponential distribution, we used *λ*_t_=(1.45·2500)^−1^ (reduced mobility width for in single cell within *M*) and *λ*_r_=1/(*μ*_r_·0.002+0.2). For the uniform distribution, we used an ellipsoid with radii *ν*_t_=0.006 and *ν*_r_=*μ*_r_·0.02+1.

We compared our adaptive EM clustering with two common clustering methods: *k*-means and DBSCAN. Since *k*-means needs a fixed number of clusters *k* and appropriate start values for the centroids, used *k*-means++ [22] for estimating good starting values and give it an advantage by assigning the true number of partitions. DBSCAN has the advantages that it does not need to know the number of clusters in advance and that it can find non-linear cluster boundaries, but it does not easily yield parametric cluster descriptors.

To measure the quality of an obtained clustering  we use the Fowlkes-Mallows index (FMI, [23]) and the normalized variation of information (NVI) score [24].

For the FMI one considers all pairs of data points. If two data points belong to the same true partition of , they are called *connected*. Accordingly, a pair of data points is called *clustered* if they are clustered together by the clustering method are evaluating. Pairs of data points that are both connected and clustered are called true positives (TP). False positives (FP, not connected but clustered) and false negatives (FN, connected but not clustered) are computed analogously. The FMI is the geometric mean of precision and recall: $\text {FMI}(\mathcal {P}, \mathcal {C}) := \sqrt {TP / (TP + FP) \cdot TP / (TP + FN)}$, where  is the true partition and  is the clustering. We have $\text {FMI}(\mathcal {P}, \mathcal {C}) \in [0, 1]$, and $\text {FMI}(\mathcal {P}, \mathcal {C}) = 1$ indicates perfect agreement. The FMI is difficult to interpret when the number of clusters in  and  differs significantly.

Therefore we use a second measure that considers cluster sizes only, the normalized variation of information (NVI). To compute the NVI, an auxiliary ($|\mathcal {P}| \times |\mathcal {C}|$)-dimensional matrix *A*=(*a*_*i*,*j*_) is computed, where *a*_*i*,*j*_ is the number of data points within partition *i* that are assigned to cluster *j*. The NVI score is defined via entropies; let *n* be the number of data points and

$$\begin{aligned} H(\mathcal{P}) &:= -\sum_{i \leqslant |\mathcal{P}|} \frac{\sum_{j \leqslant |\mathcal{C}|} a_{i,j} }{n} \log \frac{\sum_{j \leqslant |\mathcal{C}|} a_{i,j} }{n}, \\ H(\mathcal{C}) &:= -\sum_{j \leqslant |\mathcal{C}|} \frac{\sum_{i \leqslant |\mathcal{P}|} a_{i,j} }{n} \log \frac{\sum_{i \leqslant |\mathcal{P}|} a_{i,j} }{n}, \\ H(\mathcal{P} | \mathcal{C}) &:= -\sum_{j \leqslant |\mathcal{C}|} \sum_{i \leqslant |\mathcal{P}|} \frac{a_{i,j}}{n} \log \frac{a_{i,j}}{\sum_{i' \leqslant |\mathcal{P}|}a_{i',j}}, \\ H(\mathcal{C} | \mathcal{P}) &:= -\sum_{j \leqslant |\mathcal{C}|} \sum_{i \leqslant |\mathcal{P}|} \frac{a_{i,j}}{n} \log \frac{a_{i,j}}{\sum_{j' \leqslant |\mathcal{C}|}a_{i,j'}}, \\ N\!V\!I(\mathcal{P}, \mathcal{C}) &:= \left\{ \begin{array}{lr} \frac{H(\mathcal{P} | \mathcal{C}) + H(\mathcal{C} | \mathcal{P})}{H(\mathcal{P})} & \text{if} \,\, H(\mathcal{P}) \neq 0, \\ H(\mathcal{C}) & \text{otherwise.} \end{array} \right. \end{aligned} $$

Here $NVI(\mathcal {P}, \mathcal {C}) = 0$ indicates perfect agreement between the cluster size distributions. Together, an FMI of 1 and an NVI score of 0 indicate a perfect clustering.

**Figure 4:**
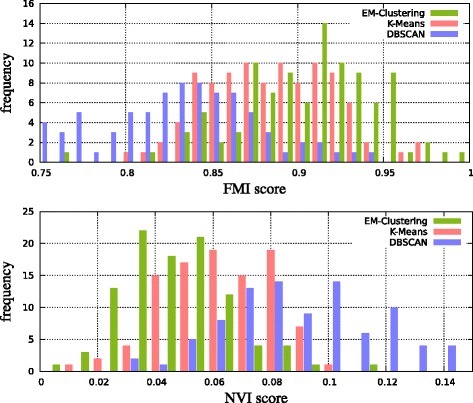
Histograms of Fowlkes-Mallows index (FMI; higher is better) and normalized variation of information (NVI; lower is better) comparing 100 simulated measurements containing partitioned peak locations with their clusters produced by the different methods.

For the first test, we evaluated 100 sets of data points distributed as described above. The cluster model (normal, exponential or uniform) was drawn randomly. The results show that even with the advantage that *k*-means knows the true *k*, our adaptive EM clustering performs best on average in terms of FMI and NVI score (Figure 4).

**Figure 5:**
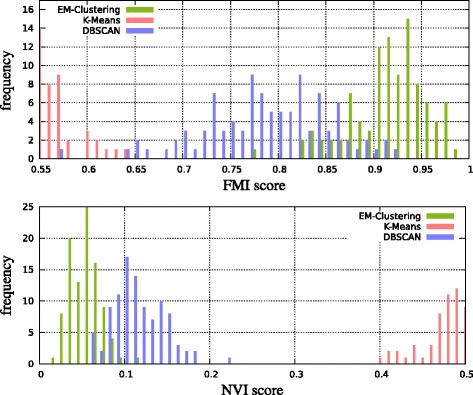
Histograms similar to Figure 4, but in a more realistic noisy scenario (see text). An FMI of 1 and NVI of 0 would be optimal.

For the second test, we additionally inserted 200 uniformly distributed (noise) peaks into the measurement area. All these peaks are singletons and have no matching peaks. The results (Figure 5) show that the adaptive EM clustering still performs best on average, whereas *k*-means fails.

### Comparison of automated online peak extraction with manual offline annotation

The fourth experiment compares extracted peaks from a time series of measurements of two automated methods to an expert manual annotation. The automated methods are our online analysis process described here and automated peak detection using the commercial VisualNow software.

Here 15 rats were monitored in 20 minute intervals for up to a day. Each rat resulted in 30–40 measurements (a time series) for a total of 515 measurements. To track peaks over time, we used the previously described EM clustering method.

**Figure 6:**
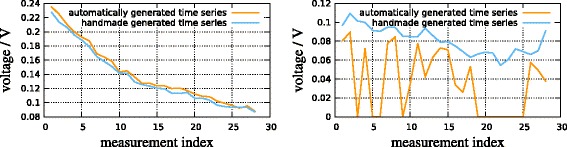
Time series of discovered intensities of two peaks. Left: A peak with agreement between manual and automated online annotation. Right: A peak where the online method fails to extract the peak in several measurements. If one treated zeros as missing data, the overall trend would still be visible.

As an example, Figure 6 shows time series of intensities of two peaks detected by computer-assisted manual annotation and using our online algorithm. The example shows that there are cases where the sensitivity of the online algorithm is not perfect; this is mainly true for peaks whose intensity only slightly exceeds the background noise.

**Figure 7:**
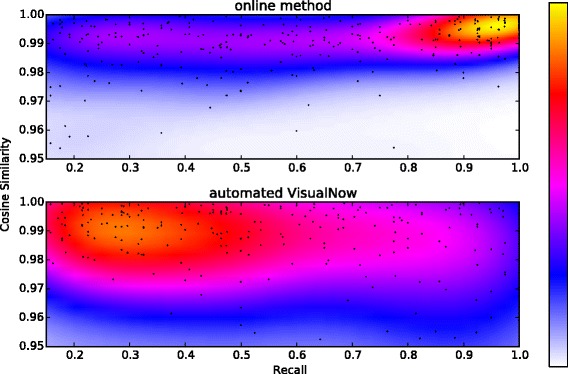
Kernel density estimation (kde) plots of recall and cosine similarity of peak intensity, comparing automatically picked peaks from our online algorithm and VisualNow against expert annotation. Each dot corresponds to a time series of one peak. Optimal results would be a recall of 1.0 and a cosine similarity of 1.0 for each time series.

To obtain an overview over all time series, we computed the cosine similarity *γ*∈ [−1,+1] time series of peak intensities discovered by manual annotation and each automated method. We also computed the recall automated method for each time series, that is, the relative fraction of measurements where the peak was found by the algorithm among those where it was found by manual annotation. Figure 7 shows overall good agreement between both automated methods (our online method and automated VisualNow peak extraction) with the expert manual annotation. The cosine similarity of the inferred time series is in better agreement than the more variable recall. When comparing automated methods against each other, we outperform VisualNow in terms of sensitivity and computation time: About 31*%* of the points extracted by the online method exceed 90*%* recall and 98*%* cosine similarity whereas only 5*%* of the time series extracted by VisualNow achieve these values. The peak detection of one measurement takes about 2 seconds on average (when the whole measurement is available at once) with the online method and about 20 seconds with VisualNow on the desktop computer described above. VisualNow only provides the position and signal intensity of the peak’s maximum, whereas our method additionally provides shape parameters.

Problems of our online method stem from low-intensity peaks only slightly above the detection threshold, and resulting fragmentary or rejected peak chains.

## Discussion and conclusion

We presented the first approach to extract peaks from MCC/IMS measurements while they are being captured, with the long-term goal to remove the need for storing full measurements before analyzing them in small embedded devices. Our method is fast and satisfies the time restrictions even on a low-power CPU platform like a Raspberry Pi and outperforms existing software.

While performing well on single spectra, there is room for improvement in merging one-dimensional peak models into two-dimensional peak models. Our method has to be further evaluated in clinical studies or biotechnological monitoring settings. It also has not been tested with the negative mode of an IMS for lack of data. In general, the robustness of the method under adversarial conditions (high concentrations with formation of dimer ions, changes in temperature or carrier gas flow in the MCC) has to be evaluated and probably improved.

## Appendix A: peak descriptors and parameters

The shifted Inverse Gaussian distribution with parameters *o* (shift or offset), *μ* (mean minus shift, also called relative mean) and *λ* (shape) is given by (1). There is a bijection [13] between (*μ*,*λ*,*o*) and the descriptors (*μ*^′^,*σ*,*m*), which are the absolute mean *μ*^′^=*o*+*μ*, the standard deviation *σ* and the mode *m*. Given (*μ*,*λ*,*o*), we have

$$\begin{array}{*{20}l} \mu' &= \mu + o, \\ \sigma &= \sqrt{\mu^{3} / \lambda}, \\ m &= \mu \cdot \big(\sqrt{1 + (9\mu^{2})/(4\lambda^{2})} - (3\mu)/(2\lambda) \big) + o, \end{array} $$

and, given (*μ*^′^,*σ*,*m*), we use auxiliary expressions *p* and *q* to find

$$\begin{array}{*{20}l} p &:= \big(-m (2 \mu' + m) + 3 \cdot (\mu^{'2} - \sigma^{2})\big) / \big(2 (m - \mu')\big),\\ q &:= \big(m (3 \sigma^{2} + \mu' \cdot m) - \mu^{'3}\big) / \big(2 (m - \mu')\big), \\ o &= -p/2 - \sqrt{p^{2}/4 - q}, \\ \mu &= \mu' - o, \\ \lambda &= \mu^{3} / \sigma^{2}. \end{array} $$
